# Why didn’t the senior citizen cross the road? Gait speed in community-dwelling older adults with mobility limitations relative to pedestrian crossing times

**DOI:** 10.1093/ageing/afaf345

**Published:** 2025-12-08

**Authors:** Max J Western, Janet Withall, Rory Sheppard, Colin Greaves, Melvyn Hillsdon, Afroditi Stathi

**Affiliations:** Department for Health, University of Bath, Bath, UK; School of Sport, Exercise and Rehabilitation Sciences, University of Birmingham, Birmingham, UK; College of Biomedical and Life Sciences, Cardiff University, Cardiff, UK; School of Sport, Exercise and Rehabilitation Sciences, University of Birmingham, Birmingham, UK; Sport and Health Sciences, University of Exeter, Exeter, UK; School of Sport, Exercise and Rehabilitation Sciences, University of Birmingham, Birmingham, UK

**Keywords:** gait speed, mobility, built environment, physical activity, community dwelling, ageing, older people

## Abstract

**Background:**

Supporting older adults to maintain physical activity is a key tool in combating the rising prevalence of physical frailty and its associated social, health and economic burden. Walkability of the physical environment, which in urban areas includes pedestrian infrastructure and safety, is a key facilitator of physical activity. Road crossings may be one such factor that impacts walkability for older populations.

**Objective:**

This study aimed to determine what proportion of community dwelling adults ≥65 years of age classified as frail/pre-frail in terms of their lower limb physical function would meet the 1.2 m/s required walking speed for pedestrian crossings, and the demographic characteristics associated with low gait speed.

**Methods:**

Four-metre walking speed data from 1110 older adults in two randomised controlled trials were analysed. Gait speed and the proportion meeting the 1.2 m/s ‘green signal’ crossing time was calculated. Generalised linear models explored differences in gait speed by sex, age, education, marital status, socioeconomic status and physical function.

**Results:**

Seventeen (1.5%) of the sample walked fast enough at their usual comfortable pace to cross during a 1.2 m/s green signal. The mean gait speed for the sample was 0.77 (SD 0.19). Older age, lower physical function and non-white ethnicity were characteristics associated with slower gait speed.

**Conclusions:**

Pedestrian crossings assuming a gait speed of 1.2 m/s are inadequate for mobility-limited older adults to cross at a comfortable walking speed. Further studies are needed to assess the impact of slow gait speed on outdoor physical activity and to develop policies for more accommodating pedestrian infrastructure.

## Key points

- Little is known about the gait speed profile of a growing sub-population of mobility limited older adults.
- Typical road crossing times assume a gait speed of 1.2 m/s to cross during the green pedestrian signal.
- Just 17 (1.5%) of our sample of 1110 frail/pre-frail community dwelling older adults would meet this gait speed comfortably.
- The average gait speed was 0.77 m/s, and slower speed was associated with worse physical function and non-white ethnicity.

## Background

Regular physical activity reduces the risk of physical frailty, falls, cognitive decline, dementia, cardiometabolic diseases, healthcare use and loss of independence in adults aged over 65 years [1, 2]. Socioecological factors like self-confidence, attitudes, social support and norms and the physical environment influence both motivation and ability to stay active in later life [3]. Two recent systematic reviews highlight the importance of walking infrastructure and community participation in promoting physical activity among UK older adults [4, 5]. For older people who are already facing some degree of mobility limitations, demanding environmental features can significantly hinder physical activity. With frailty affecting an estimated 40% of UK adults aged over 65, and 90% of over 85 years [6, 7], it is crucial to create supportive environments to reduce the risk of mobility disability in this population.

Studies have reported that pedestrian crossings, which typically assume a gait speed of 1.2 m/s, could act as a deterrent to outdoor physical activity in older populations [8–10]. Asher and colleagues demonstrated a normal average gait speed of 0.9 m/s for males and 0.8 m/s for females in a sample of 3145 over 65 year olds recruited from the general UK population, with only 16% and 7% respectively walking fast enough to meet the 1.2 m/s green signal [11]. Similar results were observed in a sample of 355 Irish older adults, which demonstrated a linear decline in gait speed from 1.3 m/s at age 60 to 0.73 m/s at age 89 [12]. A perceived inability, apprehension or fear and anxiety towards road crossing are some identified consequences of slower gait speed [13]. Little is currently known about the gait speed of community-dwelling people with limited mobility, particularly in relation to pedestrian crossing time requirements.

This short report aims to determine what proportion of community-dwelling, mobility-limited older adults in the UK have a normal gait speed sufficient to meet the pedestrian crossing requirement of 1.2 m/s. Additionally, we examine the characteristics associated with slower gait speed in this population.

## Methods

This descriptive cross-sectional study includes 1110 older adults who took part in two large randomised controlled trials aimed at supporting people over 65 with mobility limitations and not in full-time employment. To accurately assess normal gait speed, only baseline (pre-intervention) data from these trials are used in the analysis.

### Participants

This data draws from the baseline assessment of physical function in the REtirement in ACTion (REACT) study [14] that recruited 777 (514 female) older adults, and the Active, Connected, Engaged (ACE) study [15] that recruited 528 (345 female) older adults. Participants in both trials were recruited from multiple UK sites based on having a Short Physical Performance Battery (SPPB) score between 4 and 9 inclusive [16]. The samples reflected the wider UK population in terms of age, ethnicity and index of multiple deprivation, but included fewer males and adults aged 65–69 (see Table 3 in [17]). Both studies received NHS Research Ethics Committee approval (REACT: 15/LO/2082; ACE: 21/LO/0433). Of the 1305 recruited across both trials, data from 1110 was included in this analysis due to the omission of participants whose gait speed times were not recorded (*n* = 195).

### Gait speed analysis

The 4-m gait speed component of the SPPB was used to measure participants ‘normal comfortable walking speed’ from a standing start [18]. Participants performed the walk twice in their assessment, with the fastest velocity (m/s) of the two calculated to represent their normal gait speed. Descriptive statistics were used to determine the average walking speed of the cohort, and the proportion of participants who would meet the 1.2 m/s assumed pedestrian crossing speed [11]. Correlations (for continuous outcomes) and generalised linear models using the identity link function and controlling for other listed demographic variables as covariates (for categorical outcomes) explored variation in gait speed associated with a range of demographic characteristics including physical function, sex, age, marital status and ethnicity and socioeconomic position (Table 1).

**Table 1:** Unadjusted mean (SD) gait speed scores according to characteristics.

**Category**	***N* (%)** ^**a**^	**Mean (SD)** **Gait speed (m/s)**	**Difference** ^**b**^
**Total sample**	**1110**	**0.77 (0.19)**	
Sex			*P* = .117
Male	397 (36%)	0.79 (0.19)	
Female	713 (64%)	0.76 (0.19)	
Age			*P* = .057
65–74	403 (37%)	0.81 (0.19)	
75–84	518 (46%)	0.76 (0.18)	
85+	185 (17%)	0.73 (0.18)	
Ethnicity			*P* < .001
White British/-Irish/-other	1053 (95%)	0.78 (0.18)	
Non-white	56 (5%)	0.66 (0.19)	
Marital status			*P* = .139
Single, widowed or divorced	540 (55%)	0.75 (0.18)	
Married or cohabiting	450 (45%)	0.79 (0.19)	
Highest education attainment			*P* = .103
Secondary education	506 (46%)	0.75 (0.18)	
College or vocational qual	471 (42%)	0.80 (0.19)	
First degree or higher	130 (12%)	0.77 (0.20)	
Index of multiple deprivation			*P* = .195
Decile 1–3	269 (24%)	0.75 (0.18)	
Decile 4–6	333 (30%)	0.78 (0.18)	
Decile 7–10	507 (46%)	0.77 (0.20)	
SPPB total score			*P* < .001
4–7 (frail)	529 (48%)	0.66 (0.15)	
8–9 (pre-frail)	581 (52%)	0.87 (0.15)	

^a^The sample size for demographic categories does not always add up to 1110 due to missing data of a given variable; percentages represent the proportional representation of available data.

^b^To isolate the effects of dependent variables, all other variables in the table were entered as covariates in each model at either the continuous level (age, IMD and SPPB) or categorical level (sex, education, ethnicity and marital status).

## Results

**Figure 1:**
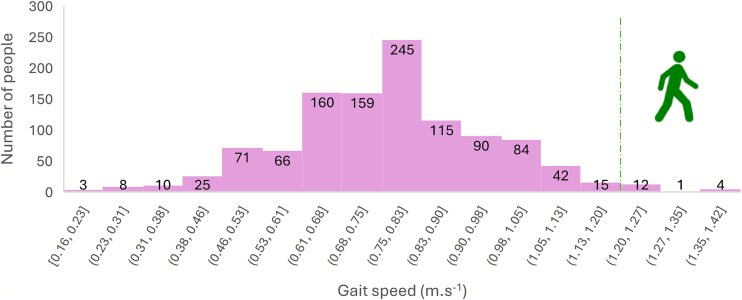
Distribution of gait speed scores within the sample (*n* = 1110).

Table 1 displays the demographic characteristics of the sample along with the mean (SD) gait speeds in each category. Figure 1 presents the distribution of gait speed scores across the sample. The overall mean gait speed for the whole sample was 0.77 (SD: 0.19)m/s. Only 17 (1.5%) of the 1110 sample had a walking speed of at least 1.2 m/s, with 136 (12%) walking faster than 1.0 m/s, and 502 (45%) walking faster than 0.8 m/s at their normal comfortable speed.

Gait speed was significantly negatively correlated with age (r = −0.150, 95% CI: −0.207 to −0.092, *P* < .001) and significantly positively correlated with total physical; function score (r = 0.648, 95% CI: 0.587–0.659, *P* < .001) but not with Index of Multiple Deprivation decile (r = 0.058, 95% CI: −0.001 to 0.116, *P* = .055). When controlling for covariates, those with lower baseline physical function and non-white ethnicity had significantly lower gait speed than people with higher physical function and white (British or non-British) ethnicity, respectively. Pairwise comparisons also revealed that people aged 65–74 years were significantly quicker by an estimated 0.025 m/s than those aged 75–84 years (*P* = .018), and that those educated to secondary school level were estimated to be −0.021 m/s slower than those who attended or completed college (*P* = .035). These data can be interpreted in light of a minimally clinically important difference in gait speed of 0.10–0.20 m/s [19].

## Discussion

In a geographically dispersed and representative sample [17] of community dwelling older adults with some degree of mobility limitation the average gait speed of 0.77 m/s is markedly slower than that needed to cross a pedestrian crossing during the green signal (1.2 m/s). A green signal on a 5-m road expects road crossers to do so in 4.2 seconds, whereas an average participant in our sample would take 6.5 seconds. Only 1.5% (or 17 out of 1110) of our sample had a comfortable walking speed sufficient to cross in time and that is not accounting for participants who, for example, may be fatigued after a longer walk, carrying groceries or navigating varied or uneven terrains. Older age and worse overall physical function were associated with slower gait speed, as was non-white ethnicity—though this group made up a small proportion of our sample. These findings highlight populations that may be especially disadvantaged by current pedestrian crossing standards and warrant further study in greater numbers. Notably, over half of participants would need to increase their gait speed by 50% or more to cross safely within the designated time.

Given the sample’s mobility profile, it is not surprising we observed slower walkers than other studies on pedestrian crossing time [11, 20]. However, it is worth noting that this mobility profile (i.e. SPPB <9) applies to around 35% of the UK’s over-70s population [21], a population who would benefit from an environment that instils confidence in rather than undermining walking capability [22]. Concerns about completing a pedestrian crossing or forcing people to walk faster than their comfortable walking pace may lead to an increased risk of injuries or falls or a reduction in confidence to get out and about [13, 23]. Reduced outdoor mobility would likely lead to reductions in community engagement, increased risk of social isolation and loneliness, and a quicker transition to severe frailty [24]. Low gait speed, even when controlling for performance in other SPPB domains (strength and balance), is associated with increased risk of inability to do activities of daily living and falling [25]. Given that this pre-frail to frail population are vulnerable to such adverse outcomes [6], environments that facilitate walking behaviours of people with mobility limitations should be a priority for public health policy.

We acknowledge the range of efforts to make road crossings safer in parts of the UK, such as the use of smart crossings and sensors, which hold traffic, if not the green signal, until the road is clear, and count down timers to help guide crossers on wider roads [26]. Such innovations are likely to reduce accidents at pedestrian crossings, but without diligent public awareness campaigns, particularly directed at the older adults with mobility limitations, they may do little to encourage and empower such people to get out and about. A more useful change, also proposed in 2015 [27], would be to ensure that the reassuring green signal stays present for a time that community dwelling frail or pre-frail older people can cross without undue haste. Based on our data, assuming a lower walking speed of 0.7 m/s, implementing a green signal duration of ~7-seconds for a 5-m road crossing (rather than 4.2-seconds), would be a good place to start.

There are some limitations to this study that should be considered when interpreting the findings. When using the SPPB, the fastest time from the two 4-m gait assessments is taken as the valid assessment of ‘normal gait speed’. This may therefore overestimate participants’ usual, comfortable walking pace. Moreover, participants gait speed was measured in favourable rested conditions, indoors, and thus not entirely representative of a road crossing. While these factors may underestimate the problem, we are not able to ascertain if ‘normal walking speed’ would be judged the same outdoors at a road crossing as was in our indoor study assessment. Experimental research has shown using a 10-m walk test that gait characteristics such as stride length, speed and symmetry is significantly lower indoors compared to outdoor conditions [28]. Additionally, because this is a cross-sectional analysis, the observed associations and suggested implications of slower gait speed should not be interpreted as causal. Further research is needed to examine how slow gait speed affects walking attitudes, self-efficacy, physical activity and the risk of accidents or injuries during road crossings among older adults.

Nonetheless, these findings underscore an important opportunity for policymakers and practitioners in transport and urban planning to create more accessible walking environments for older adults. While promoting physical activity and improving function remain vital for reducing frailty, even modest policy changes—such as extending pedestrian crossing times by just a few seconds—could meaningfully enhance mobility and independence for this growing population. We encourage stakeholders to consider these adjustments to better support older adults in maintaining active, independent lives.

## Data Availability

The dataset used for analysis can be accessed by contacting the corresponding author.
